# Mesenteroaxial Gastric Volvulus: A Report of a Rare Case

**DOI:** 10.7759/cureus.87559

**Published:** 2025-07-08

**Authors:** Jose Oswaldo Ferre Bello, Carlos Iskyam Zaldo Arredondo, Claudia Beatriz León González, Karla Andrea Muñoz Rodríguez

**Affiliations:** 1 General Surgery, Hospital General de León, León de Los Aldama, MEX

**Keywords:** gastric volvulus, intestinal occlusion, mesenteroaxial, tomography, untwisting

## Abstract

Gastric volvulus is a rare surgical emergency characterized by the rotation of the stomach by more than 180° along one of its axes, which can result in significant upper gastrointestinal obstruction. If not promptly treated, it can lead to severe complications and carries a considerable risk of morbidity and mortality. Clinically, patients typically present with epigastric pain, nausea, and vomiting of gastrointestinal contents. Diagnosis is primarily based on the patient’s history and clinical presentation, with confirmation through imaging studies. Definitive treatment is surgical. We report the case of a 74-year-old male who presented to the emergency department with a 48-hour history of intestinal obstruction. Imaging confirmed a mesenteroaxial gastric volvulus, which required emergency surgical intervention. The patient had an uneventful postoperative recovery.

## Introduction

Gastric volvulus is defined as the rotation of the stomach by more than 180° along one of its axes, leading to high intestinal obstruction [1-5]. It is considered a rare surgical emergency, most frequently reported in pediatric patients due to congenital diaphragmatic defects [2,6]. However, cases have also been described in adults, where it is primarily associated with type II-III hiatal hernias [3,6], with peak incidence occurring in the fifth decade of life [6]. Currently, the condition is associated with a high morbidity and mortality rate, which may reach up to 80% [3].

Historically, gastric volvulus has been infrequently described. It was first reported in 1579 as a complication of a strangulated diaphragmatic hernia and later, in 1866, as a cause of death due to intestinal obstruction. The first successful surgical intervention was documented in 1896 [1,6].

Gastric volvulus is primarily classified based on its etiology into primary and secondary forms [3,4,6], and anatomically into organoaxial, mesenteroaxial, and mixed types [1,4-6]. The organoaxial type, where the stomach rotates along its long axis, accounts for 60-66% of cases. The mesenteroaxial type, seen in 30-33% of cases, involves rotation along the short axis, often resulting in the antrum being displaced above the gastroesophageal junction [4-6].

Clinically, gastric volvulus can present in acute or chronic forms [4]. Patients typically experience thoracoabdominal colicky pain, predominantly in the epigastric region, along with nausea and vomiting of gastrointestinal contents. A classic diagnostic feature is Borchardt’s triad - epigastric pain, vomiting, and inability to pass a nasogastric tube - described in 1904 and present in approximately 70% of cases [4-6].

Diagnosis is largely clinical, based on patient history and physical findings, but confirmation is achieved through contrast-enhanced CT [6]. Although there are no specific laboratory tests for gastric volvulus, elevated serum amylase levels have been observed in some patients [7].

Surgical management focuses on detorsion of the stomach and addressing the underlying cause to prevent recurrence [1,5,8-10]. Gastropexy is recommended to secure the stomach and reduce the risk of recurrence [1,5]. Both open and laparoscopic approaches are viable, with current literature favoring the laparoscopic method.

## Case presentation

A 74-year-old male patient with no significant medical history presented to the emergency department with a 48-hour history of abdominal pain and distension, predominantly epigastric cramping, accompanied by nausea, vomiting of gastrointestinal contents, and absence of bowel movements.

On physical examination, the patient was neurologically intact and cooperative, with algid facies, underhydrated mucotegumentary tissue, a distended abdomen, decreased peristalsis, and abdominal tenderness on mid and deep palpation in the left upper quadrant. Rebound was negative. A left inguinal hernia was noted, which was not painful and was reducible without difficulty.

**Figure 1:**
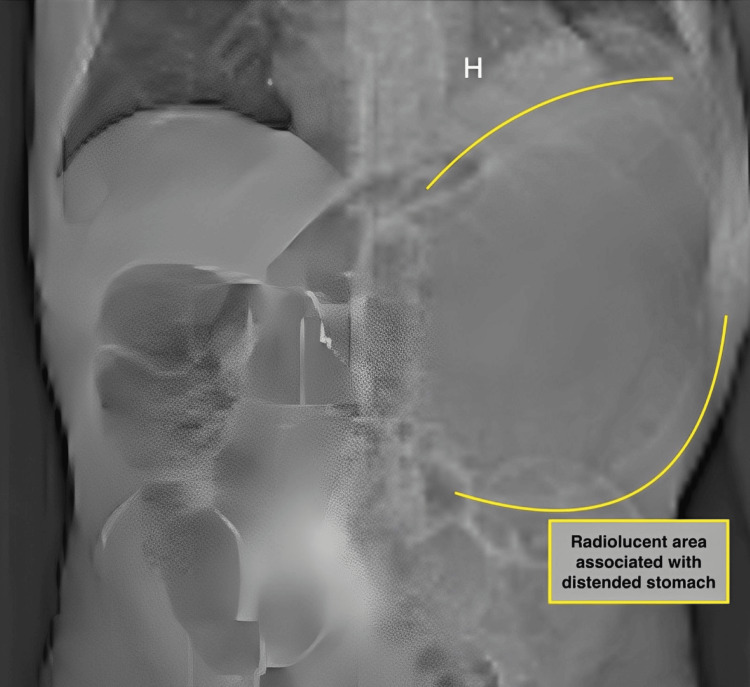
Abdominal X-ray showing a radiolucent area in the upper left quadrant corresponding to the stomach, which appears markedly distended

An abdominal X-ray was performed, revealing a large radiolucent area associated with hydro-air levels in the left upper quadrant (Figure 1).

Placement of a nasogastric tube was attempted, yielding a low gastric output of 20 cc, with no improvement in abdominal pain following insertion.

Clinical laboratory tests were requested upon admission, and the results are presented in Table 1.

**Table 1:** Summary of laboratory test results

Laboratory test	Patient value	Normal range
Glucose	139 mg/dL	74-106 mg/dL
Urea	53 mg/dL	19-43 mg/dL
Blood urea nitrogen	24.8 mg/dL	9-20 mg/dL
Creatinine	0.9 mg/dL	0.6-1.25 mg/dL
Amylase	151 U/L	30-110 U/L
Lipase	639 U/L	23-300 U/L
Sodium	138 mmol/L	137-145 mmol/L
Chloride	103 mmol/L	98-107 mmol/L
Potassium	4.2 mmol/L	3.5-5.1 mmol/L
Leukocytes	15.49 × 10³/μL	4-10 × 10³/μL
Hemoglobin	14.8 g/dL	12-16 g/dL
Platelets	325 × 10³/μL	130-400 × 10³/μL
Prothrombin time	15.1 seconds	11-15 seconds
Partial thromboplastin time	29.7 seconds	26-36 seconds
International normalized ratio	1.17	-

**Figure 2:**
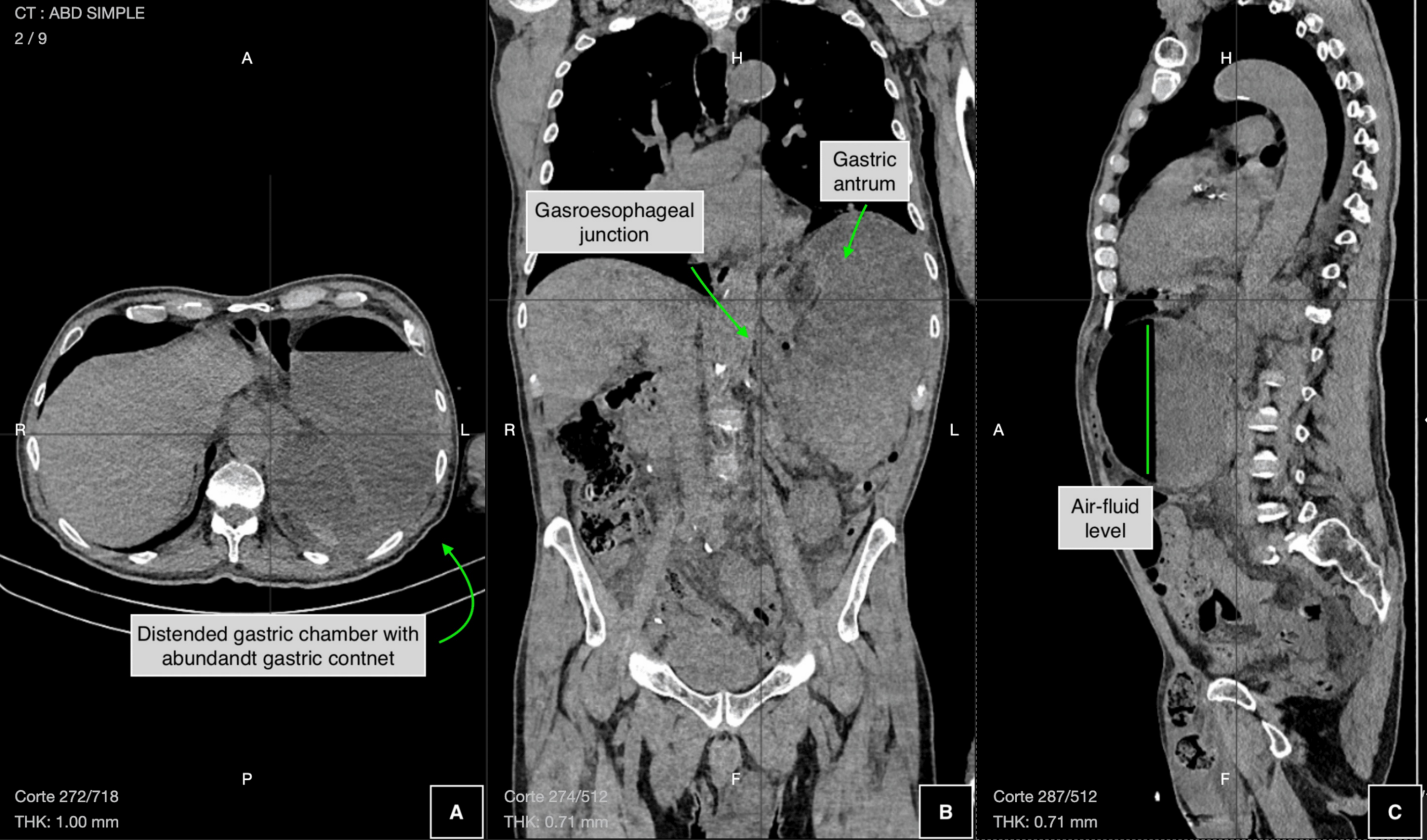
Abdominal CT scan showing mesenteroaxial gastric volvulus (A) Distended gastric chamber. (B) Displacement of the antrum above the gastroesophageal junction. (C) Air-fluid level secondary to gastric obstruction caused by mesenteroaxial volvulus.

An abdominal CT scan was performed, revealing an overdistended, fluid-filled stomach with rotation of the gastric antrum above the gastroesophageal junction. The duodenum, small intestine, and colon showed normal positioning and densities, although a decreased intestinal lumen diameter was noted. These radiological findings were consistent with mesenteroaxial gastric volvulus (Figure 2).

**Figure 3:**
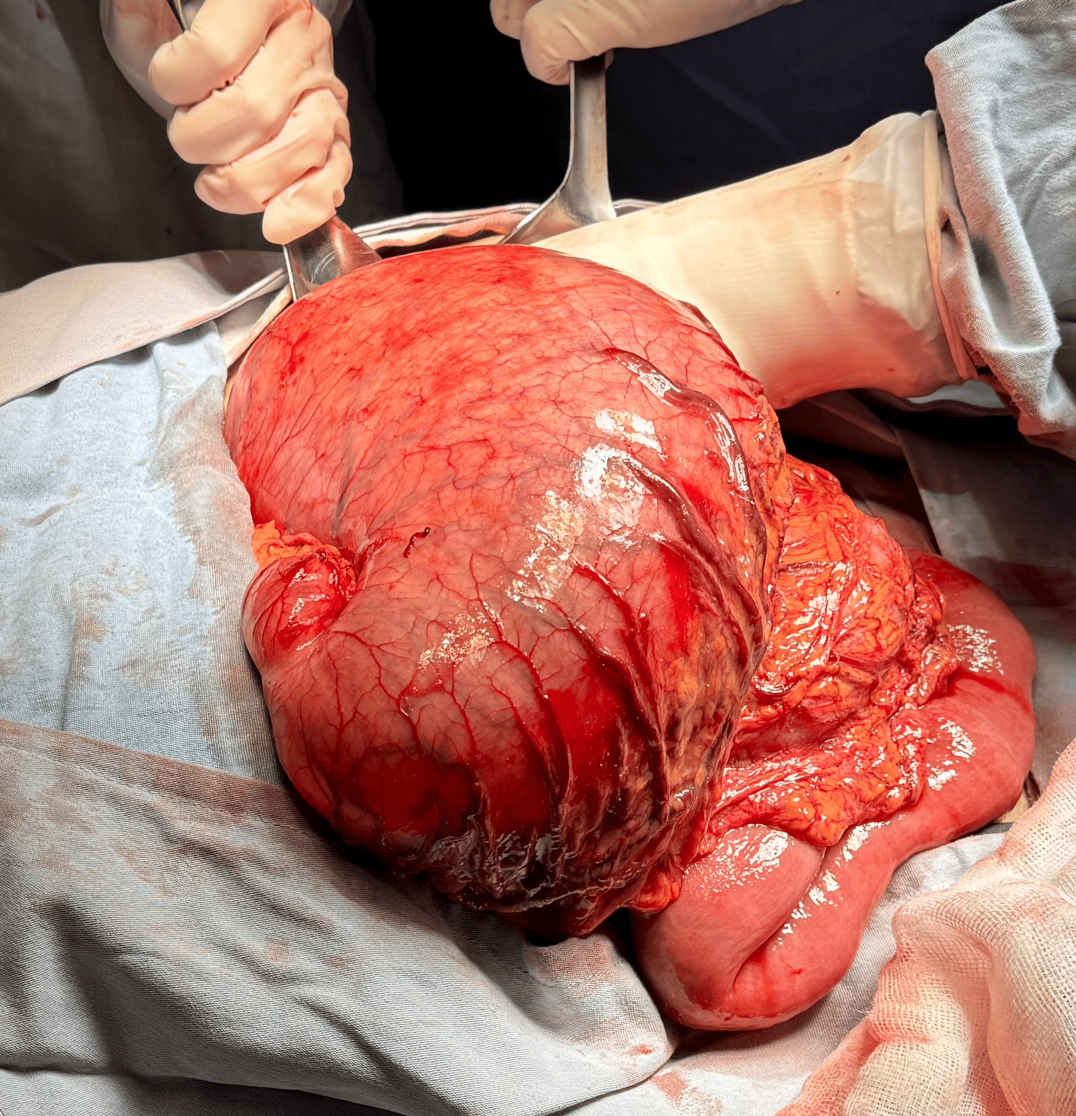
Intraoperative view showing gastric distension associated with volvulus The gastric tissue appears viable, with no discoloration or signs of perforation.

Surgical management was proposed and accepted by the patient. An exploratory laparotomy was performed, revealing significant gastromegaly associated with mesenteroaxial gastric volvulus. Manual untwisting of the stomach was carried out, and gastric contents were aspirated. Tissue inspection revealed no discoloration or signs of perforation. A gastropexy was performed, anchoring the fundus to the diaphragm and the anterior aspect of the stomach to the abdominal wall using simple 3-0 multifilament sutures. Hemostasis was verified, and the abdominal wall was closed, completing the surgical procedure (Figure 3).

The patient was transferred to post-anesthesia recovery and subsequently admitted for monitoring, with adequate clinical progress. He began ambulating 12 hours after surgery and passed gas at 24 hours postoperatively. An enteral diet was initiated, which he tolerated well. The nasogastric tube was removed, and he was discharged home 48 hours after the procedure. Follow-up continued through the outpatient clinic, and he was officially discharged from care five months after the initial event.

## Discussion

Gastric volvulus is associated with high morbidity and mortality [3,7], as delays in diagnosis and treatment can lead to gastric necrosis and perforation [4-6], resulting in severe and life-threatening complications. Although it has been primarily described in pediatric patients due to its common congenital origin [2], adults are not exempt from developing this condition. This underscores the importance of timely identification and management, as early diagnosis and intervention have been shown to significantly reduce morbidity and mortality, from 80% to as low as 16% [7].

In the case presented, the patient, an individual in his eighth decade of life, was diagnosed with intestinal obstruction secondary to mesenteroaxial gastric volvulus. According to the literature reviewed, this presentation is exceedingly rare, as most documented cases involve pediatric patients and the organoaxial type of volvulus [4].

This patient exhibited typical signs of intestinal obstruction with abdominal symptoms, although adult patients may also present with chest pain, as hiatal hernia is the primary cause of gastric volvulus in this age group [3-5]. Additionally, the patient presented with Borchardt’s triad, which is considered diagnostic for gastric volvulus [3-6].

Diagnosis in this case was based on clinical presentation and confirmed through abdominal CT, which revealed a mesenteroaxial gastric volvulus [5]. Although contrast studies such as upper gastrointestinal series can achieve nearly 100% sensitivity [4], in emergency settings, delays in diagnosis and management may lead to catastrophic outcomes.

Surgical management is indicated for all patients with this condition [1]. In cases secondary to diaphragmatic hernia, anti-reflux surgery should also be considered [1,9]. Literature supports exploratory laparotomy as the preferred approach in emergency scenarios [6], although laparoscopic surgery is a viable option when resources and expertise are available [3,9].

While endoscopic untwisting may be attempted, it carries a high recurrence rate [3,5]. To minimize recurrence, gastropexy is recommended [1,5], and was performed in this patient following reduction of the volvulus.

## Conclusions

Gastric volvulus is a rare condition, but it holds significant relevance in clinical practice and highlights the importance of awareness among general surgeons to ensure quality patient care. It should be considered as a potential cause of intestinal obstruction, particularly when there are signs of upper gastrointestinal obstruction. Recognizing Borchardt’s triad can support a well-founded diagnostic suspicion, guiding timely clinical decisions regarding emergency surgical intervention. A delayed diagnosis can lead to severe complications and a poorer prognosis.

Surgical management may occur in two stages: initial detorsion followed by correction of the underlying cause. This staged approach is often chosen when a single, extensive procedure is deemed too aggressive given the patient’s condition. In this case, due to the emergent nature of the presentation, an open surgical approach was selected and performed without complications. The patient demonstrated satisfactory postoperative recovery.
